# Performance Evaluation of a Preclinical SPECT Scanner with a Collimator Designed for Medium-Sized Animals

**DOI:** 10.1155/2022/9810097

**Published:** 2022-07-16

**Authors:** Yohji Matsusaka, Rudolf A. Werner, Paula Arias-Loza, Naoko Nose, Takanori Sasaki, Xinyu Chen, Constantin Lapa, Takahiro Higuchi

**Affiliations:** ^1^Department of Nuclear Medicine and Comprehensive Heart Failure Center, University Hospital of Würzburg, Würzburg, Germany; ^2^Division of Nuclear Medicine and Molecular Imaging, The Russell H Morgan Department of Radiology and Radiological Sciences, Johns Hopkins School of Medicine, Baltimore, MD, USA; ^3^Graduate School of Medicine, Dentistry and Pharmaceutical Sciences, Okayama University, Okayama, Japan; ^4^Nuclear Medicine, Medical Faculty, University of Augsburg, Augsburg, Germany

## Abstract

**Background:**

Equipped with two stationary detectors, a large bore collimator for medium-sized animals has been recently introduced for dedicated preclinical single-photon emission computed tomography (SPECT) imaging. We aimed to evaluate the basic performance of the system using phantoms and healthy rabbits.

**Methods:**

A general-purpose medium-sized animal (GP-MSA) collimator with 135 mm bore diameter and thirty-three holes of 2.5 mm diameter was installed on an ultrahigh-resolution scanner equipped with two large stationary detectors (U-SPECT5-E/CT). The sensitivity and uniformity were investigated using a point source and a cylinder phantom containing ^99m^Tc-pertechnetate, respectively. Uniformity (in %) was derived using volumes of interest (VOIs) on images of the cylinder phantom and calculated as [(maximum count − minimum count)/(maximum count + minimum count) × 100], with lower values of % indicating superior performance. The spatial resolution and contrast-to-noise ratios (CNRs) were evaluated with images of a hot-rod Derenzo phantom using different activity concentrations. Feasibility of *in vivo* SPECT imaging was finally confirmed by rabbit imaging with the most commonly used clinical myocardial perfusion SPECT agent [^99m^Tc]Tc-sestamibi (dynamic acquisition with a scan time of 5 min).

**Results:**

In the performance evaluation, a sensitivity of 790 cps/MBq, a spatial resolution with the hot-rod phantom of 2.5 mm, and a uniformity of 39.2% were achieved. The CNRs of the rod size 2.5 mm were 1.37, 1.24, 1.20, and 0.85 for activity concentration of 29.2, 1.0, 0.5, and 0.1 MBq/mL, respectively. Dynamic SPECT imaging in rabbits allowed to visualize most of the thorax and to generate time-activity curves of the left myocardial wall and ventricular cavity.

**Conclusion:**

Preclinical U-SPECT5-E/CT equipped with a large bore collimator demonstrated adequate sensitivity and resolution for *in vivo* rabbit imaging. Along with its unique features of SPECT molecular functional imaging is a superior collimator technology that is applicable to medium-sized animal models and thus may promote translational research for diagnostic purposes and development of novel therapeutics.

## 1. Introduction

Rabbits are essential experimental animals for biomedical research. Basic and translational scientists mainly use small rodents such as mice and rats to reduce housing and maintenance costs. However, many unique features of rabbits render this species attractive for investigations in various medical specialties, such as cardiology [1, 2], immunology [3], and microbiology [4, 5]. As a major advantage, rabbits are phylogenetically closer to humans than rodents [6]. One example is that uptake-2, one of the critical mechanisms of norepinephrine reuptake into cardiomyocytes to maintain cardiac nerve integrity, has substantially lower expression levels in human and rabbit hearts, while it is highly expressed in mouse and rats [7–9]. For instance, those superior cross-species translational abilities of rabbit myocardium may be relevant for the development and testing of novel cardiac sympathetic nerve system-targeting therapies. Furthermore, the bigger organ size is a fundamental advantage for various biomedical assays including *in vivo* imaging technologies which have limited spatial resolution [10–15].

Given the increasing demand of functional molecular imaging of animals, single-photon emission computed tomography (SPECT) has been evolved over the last decades. These include, but are not limited to 16-camera small animal SPECT scanner with modular scintillation cameras (e.g., FastSPECT II with active areas of up to 130 cm^2^) [16] or equipped with cadmium zinc telluride detectors (SemiSPECT) [17]. Despite those important technological advances in the context of small animal SPECT, imaging medium-sized animals such as rabbits using single-photon emission computed tomography (SPECT) have not been well established when compared to preclinical imaging of small rodents. In this regard, most preclinical SPECT systems have been developed exclusively for the investigation of mouse or rat experiments, for rabbits' larger bore sizes (>10 cm) are needed to ensure that the animals will adequately fit into the scanner. Often, as a substitute, human clinical SPECT cameras are used in rabbit experiments, but they have limited spatial resolution (>1 cm) and do not provide adequate image quality to delineate small organs [12, 14, 15].

Recently, dedicated medium-sized animal SPECT imaging systems with large bore multipinhole collimators on high-resolution animal SPECT systems with large stationary detectors have been introduced. Therefore, the purpose of this study is to investigate the performance of the SPECT system with phantoms and *in vivo* animal experiments using rabbits along with dynamic image acquisition, as the herein applied U-SPECT5-E/CT defines the duration and frames prospectively relative to other commercially available SPECT/CT systems.

## 2. Materials and Methods

### 2.1. Scanner System

**Figure 1:**
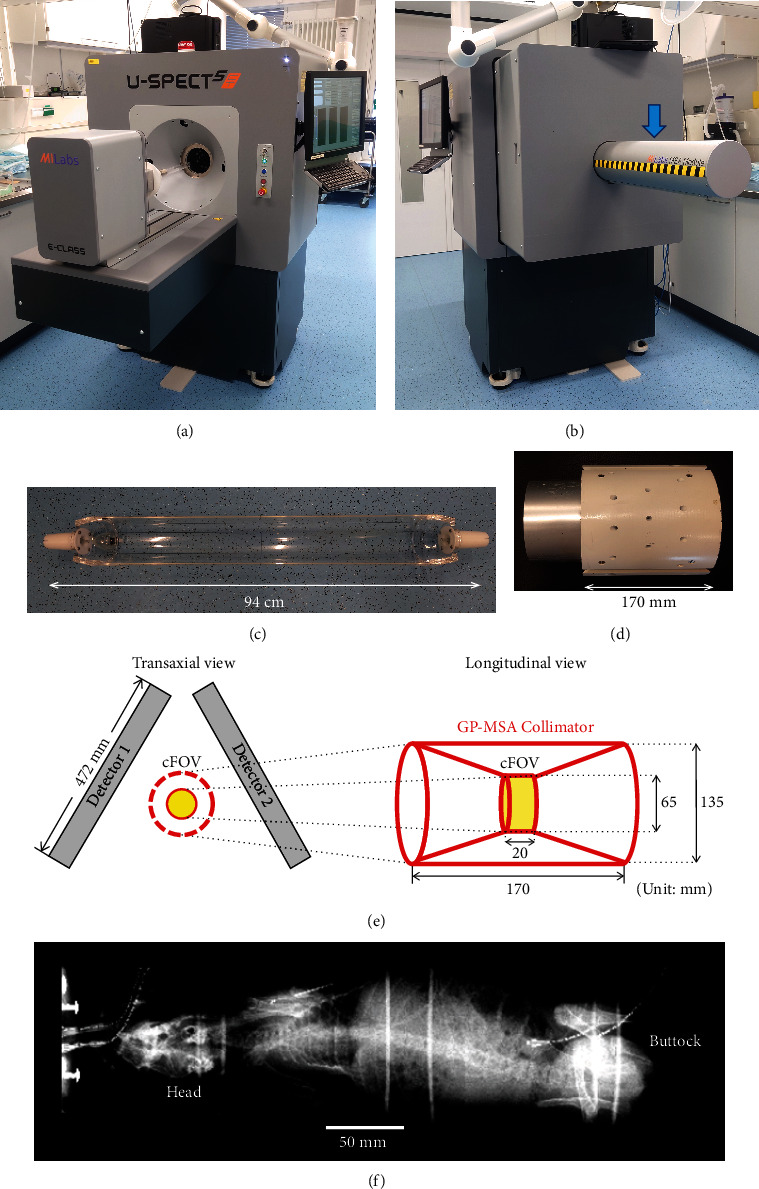
The overview of U-SPECT5/CT E-class. (a) The front side of the system. (b) The back side of the system. A tubular protrusion on the back side (blue arrow) can hold a 94 cm long bed for medium-sized animals. (c) Photo of bed for medium-sized animals. (d) General-purpose medium-sized animal (GP-MSA) collimator. (e) Geometric illustration of GP-MSA collimator in the two stationary detectors. The bore diameter of GP-MSA collimator is 135 mm, and the transaxial size of center field-of-view (cFOV) is 65 mm. (f) Scout view of a rabbit scanned with X-ray system combined with the SPECT system.

U-SPECT5/CT E-class (MILabs, Utrecht, The Netherlands) was used for SPECT imaging, displayed in Figures 1(a) and 1(b). The system performance using dedicated collimators for mice and rats had been reported elsewhere [18, 19]. Briefly, U-SPECT5-E was developed as a cost-effective substitute of the conventional U-SPECT5 system, which is also an ultrahigh-resolution SPECT system for preclinical imaging of small animals. The architecture of U-SPECT5 is based on previous generations of micro-SPECT systems (U-SPECT-II; U-SPECT+; MILabs) [20, 21] and has three stationary detectors with a size of 472 mm × 595 mm and 9.5 mm thick thallium-doped sodium iodide scintillation crystals arranged in a triangular format around the field of view (FOV). A centrally located collimator with multipinhole configuration allows the acquisition of SPECT images in a spiral step mode using an automated xyz stage [22]. To strive for cost effectiveness, U-SPECT5/CT E-class is built without the bottom detector [18, 19].

The U-SPECT5-E has a tubular protrusion on the back of the scanner (Figure 1(b)), which can be opened to hold a 94 cm long bed for medium-sized animals (Figure 1(c)). A general-purpose medium-sized animal type (GP-MSA) (Figure 1(d)) collimator has a wide bore of 135 mm diameter and 33 pinholes with 2.5 mm diameter aperture (Figure 1(e)). All the pinholes point towards a center FOV (cFOV) of 65 mm diameter and 20 mm length, which enables to acquire SPECT images of wide areas without bed movement. Due to the missing bottom detector, only the upper 22 pinholes contribute to the imaging process in the U-SPECT5-E/CT system. Since this scanner also has an X-ray irradiation capability, it can also acquire computed tomography (CT) and X-ray scout views (Figure 1(f)).

### 2.2. Image Reconstruction and Data Processing

All images were acquired in list-mode and reconstructed with the similarity-regulated ordered-subset expectation maximization (SROSEM) algorithm [23], as provided with the dedicated software (MILabs.Rec Version 8.06) of the SPECT system. This algorithm allows to avoid artifacts caused by a higher number of subsets causing potential quantification errors [24]. For scatter correction, we applied the triple energy window method [25] with a 20% photopeak window of 126 keV to 154 keV for a ^99m^Tc photopeak of 140 keV, a lower background window of 120.4 keV to 126 keV, and an upper one of 154 keV to 159.6 keV. For simplifying the reconstruction process, we always used 8 iterations with 32 subsets and a voxel size of 0.8 mm^3^. The obtained SPECT images were transferred to the AMIDE software (version 1.0.4 for Windows; open source) [26] for further analysis including Gaussian postfiltering, which was applied as recommended by Vaissier et al. [23]. This approach showed no perceptible decrease in image quality compared to the reference standard of maximum likelihood expectation maximization (MLEM) [27].

### 2.3. Performance Measurements

Sensitivity was examined using a point source with a ^99m^Tc-pertechnetate solution of 16.4 MBq. Scan time was 5 min without bed motion. Calculation was based on the National Electrical Manufacturers Association (NEMA) [28] as follows:

(1) $$\begin{array}{c} \text{Sensitivity }\left(\frac{\text{cps}}{\text{MBq}}\right)=\frac{R_{i}}{A_{\text{cal}}}, \end{array}$$

where *R*_*i*_ is the detected count rate in the photopeak window of 126 keV to 154 keV and *A*_cal_ is the total activity of the point source determined by a dose calibrator (ISOMED 2010, NUVIA Instruments, Dresden, Germany).

Uniformity was investigated using a 28 mm diameter cylindrical phantom filled homogenously with ^99m^Tc-pertechnetate solution of 20.5 MBq/mL, and the scan time was 5 min without bed motion. Gaussian postfiltering was applied with a full width at half maximum (FWHM) of 2.5 mm, equal to the resolution in the activity concentration of 1.0-29.2 MBq/mL. A cylindrical volume of interest (VOI) was placed centrally in the phantom, measuring 25 mm in diameter and 5 mm in length. Uniformity was calculated as recommended by NEMA [28] as follows:

(2) $$\begin{array}{c} \text{Uniformity }\left(\%\right)=\frac{\mathrm{Max}\text{ count}-\mathrm{Min}\text{ count}}{\mathrm{Max}\text{ count}+\mathrm{Min}\text{ count}}\times 100. \end{array}$$

In addition, a line profile was measured by placing a line passing through the center of the phantom to evaluate smooth or irregular patterns in the images.

Spatial resolution was evaluated using a hot-rod phantom whose general features are shown in the Supplementary Figure 1. The diameter of its rods ranges from 1.80 to 3.10 mm. In each section, the intercapillary distance equals the respective rod diameter of that section. The phantom was filled with a ^99m^Tc-pertechnetate solution and placed in the center of the cFOV. The cFOV of GP-MSA collimator was wide enough to cover the whole phantom without bed motion. When the activity concentration of ^99m^Tc was 29.2, 1.0, 0.5, and 0.1 MBq/mL, SPECT scanning was performed for 20 min with one bed position. Gaussian postfiltering was applied with a FWHM of 1.8 mm, corresponding the minimum rod size. In the visual analysis of the reconstructed images, the smallest discriminable rod size was determined to describe the collimator-dependent spatial resolution.

### 2.4. Phantom Image Quality

**Figure 2:**
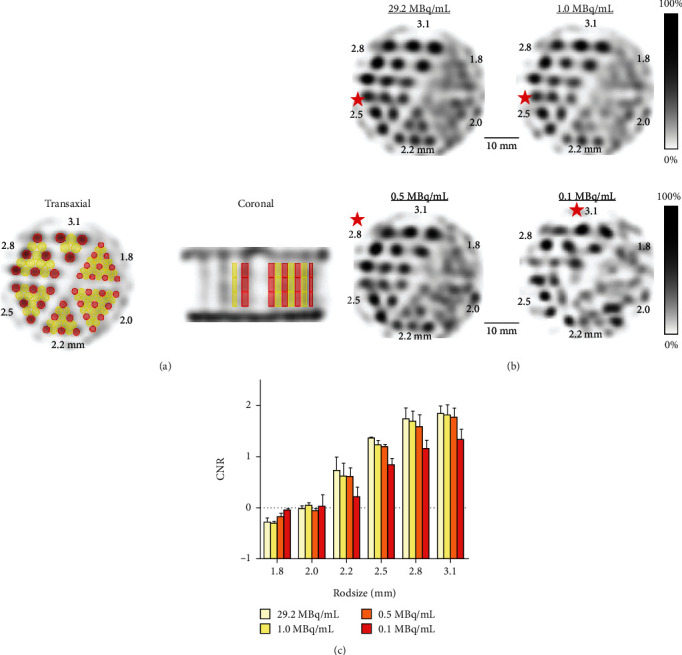
Resolution and contrast-to-noise ratios of the hot-rod phantom images. (a) Representative transaxial and coronal SPECT images of the phantom. Red and yellow cylinders indicate VOIs for hot and cold areas, respectively. (b) SPECT images of the hot-rod phantom containing 4 different activity concentrations. Slice thickness of 5.0 mm and Gaussian postfilter of full width at half maximum (FWHM) equal to minimum rod size of 1.8 mm were applied. Star marks represent the smallest rod size that can be clearly distinguished on the images of each activity concentration. (c) Contrast-to-noise ratios for the different activity concentrations. Analyzed images were optimized for each rod size by a Gaussian postfilter of FWHM equal to each diameter of the corresponding rods.

To evaluate the *in vitro* image quality, contrast-to-noise ratio (CNR) analysis was performed using the images optimized with Gaussian postfiltering for each rod size. FWHM was set to the diameter size of each investigated rod section to enhance image quality [19]. The technique for setting VOIs in the hot-rod phantom was adopted from the method described by Walker et al. [29]. Using a high-resolution computed tomography (CT) image as a template, cylindrical VOIs of 4 mm length were placed in the height center of the rods. All VOIs have a diameter of 0.9 times the size of the respective radioactive rod. In addition, VOIs of the same size as radioactive rods were placed in the nonradioactive areas in-between two radioactive rods. The same size VOIs were also place in 4 mm upper and lower slices in *z* axis as shown in Figure 2(a). The contrast *C*_*d*_ was defined as follows:

(3) $$\begin{array}{c} C_{d}=\frac{\bar{R_{d}}-\bar{B_{d}}}{\bar{R_{d}}}, \end{array}$$

where $\bar{R_{d}}$ is the mean of mean values of all radioactive VOIs for the rod size *d* and $\bar{B_{d}}$ is the mean of all nonradioactive VOIs. The noise *N*_*d*_ was defined as follows:

(4) $$\begin{array}{c} N_{d}=\frac{\sqrt{{\bar{\sigma_{R_{d}}}}^{2}+{\bar{\sigma_{R_{d}}}}^{2}}}{\bar{{\text{VOIs}}_{d}}}, \end{array}$$

where $\bar{\sigma_{R_{d}}}$ is the mean of standard deviations of all radioactive VOIs for the rod size *d* and $\bar{\sigma_{B_{d}}}$ is the mean of standard deviations of all nonradioactive VOIs. $\bar{{\text{VOIs}}_{d}}$ is the mean of mean values of both radioactive and nonradioactive VOIs for the rod size *d*.

The CNR for each size rod *d* was defined as follows:

(5) $$\begin{array}{c} {\text{CNR}}_{d}=\frac{C_{d}}{N_{d}}. \end{array}$$

The measurement for CNR was repeated three times for three VOIs in each radioactive and nonradioactive area, and the mean of three CNRs was calculated for each area.

### 2.5. *In Vivo* Cardiac SPECT Images Using GP-MSA Collimator

Animal protocols were approved by the local Animal Care and Use Committee (Regierung von Overfranken, Germany) and conducted according to the Guide for the Care and Use of Laboratory Animals [30]. During *in vivo* imaging, the animals underwent inhalation anesthesia (2.0% isoflurane, 1.5 L O_2_/min). A healthy male New Zealand White rabbit (Charles River Laboratories Inc., Sulzfeld, Germany) with 970 g body weight was placed on the dedicated bed for medium-sized animals. SPECT imaging was conducted after CT to confirm the precise position of the heart. The dynamic SPECT acquisition started just before injection of 378.5 MBq of [^99m^Tc]Tc-sestamibi (MIBI) [31] via the ear vein, with a scan time of 5 min (2 × 15 sec-frames, 1 × 30 sec-frame, and 2 × 120 sec-frames). After reconstruction, Gaussian postfiltering was applied with FWHM of 5 mm. Two oval-shaped VOIs were placed centrally in the anterior wall of the left ventricle and in the left atrium, and time activity curves were created. Static acquisition started 15 min after the injection with a scan time of 30 min. Another rabbit with 1300 g body weight was scanned in the same way to confirm the reproducibility of the results.

As a reference, the GP-MSA collimator imaging with a male Wistar rat (Charles River Laboratories Inc., Sulzfeld, Germany) with 250 g body weight was conducted after [^99m^Tc]Tc-MIBI (200 MBq) administration via tail vein. Twenty-five minutes after injection, static SPECT imaging was conducted with an acquisition time of 10 min following CT acquisition. Gaussian postfiltering was not applied. Fusion images of CT and SPECT were created with software solutions such as AMIDE [26].

## 3. Results

### 3.1. Sensitivity and Uniformity Measurements

**Figure 3:**
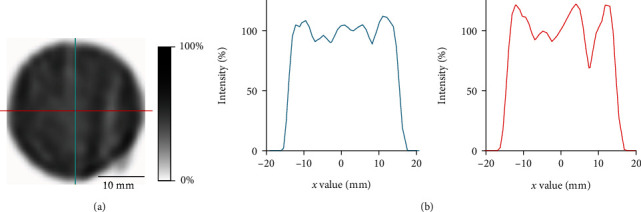
Representative image and line profiles of the cylinder phantom for uniformity. (a) Transaxial SPECT image. The activity concentration was 20.5 MBq/mL. Slice thickness of 5.0 mm and Gaussian postfilter full width at half maximum (FWHM) equal to maximum resolution of 2.5 mm were applied. (b) Line profiles of the vertical and horizontal lines on the left SPECT image. Blue and red curves are corresponding to the blue vertical and red horizontal lines.

Sensitivity of the point source was 790 cps/MBq (0.079%). Uniformity for the GP-MSA collimator was 39.0% in accordance with the NEMA protocol.  Figure 3 illustrates the representative reconstructed images for uniformity and line profiles. The profile of horizontal lines appears to be slightly more irregular than that of vertical lines.  Figure 2(b) provides the SPECT images of the hot-rod phantom used for spatial resolution analysis. In visual analysis, increased image noise was noted for lower activities. A minimum diameter of 2.5 mm clearly discriminated even in 1.0 MBq/mL, thereby representing spatial resolution. On the other hand, a discrimination of rods of 2.0 mm and smaller was not possible, even when the highest activity concentration was applied.

### 3.2. Phantom Image Quality

Figure 2(c) shows the results of CNR analysis of the six rod sizes for four different activity concentrations. The CNRs of the rod size 2.5 mm were 1.37, 1.21, 1.24, and 0.79 for activity concentration of 29.2, 1.0, 0.5, and 0.1 MBq/mL, respectively. For all activity concentrations, CNR improved continuously with increasing rod size. Although the CNR of higher activity concentrations tended to be higher in rod sizes over 2.2 mm, the tendency disappeared in rod sizes of 2.0 mm and smaller. CNR values of a rod size of 2.0 mm and smaller were almost negative for all activity concentrations. A CNR of 1.30 was achieved for over 0.5 MBq/mL at rod sizes of more than 2.5 mm and for over 0.1 MBq/mL at rod sizes of more than 2.8 mm.

### 3.3. Dynamic Myocardial Perfusion SPECT Images of Rabbits and Rats

**Figure 4:**
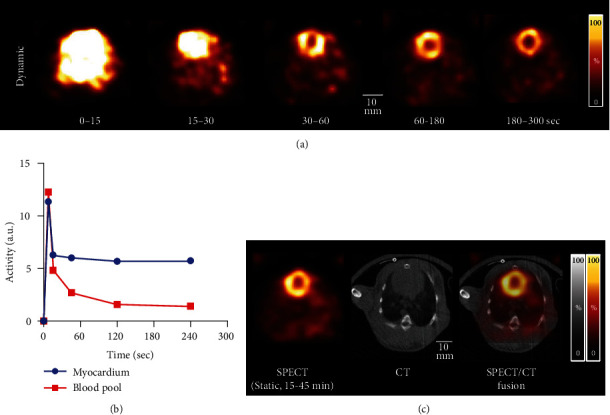
Dynamic [^99m^Tc]Tc-MIBI SPECT data of a rabbit's thorax. (a) Transaxial dynamic SPECT images at the level of the heart during 5 min after intravenous injection. (b) Time activity curves of the myocardium and blood pool during a 5 min dynamic scan. a.u.: arbitrary unit. (c) Fusion image at the (CT-based) level of the myocardium and static SPECT image during 15-45 min after tracer injection.

**Figure 5:**
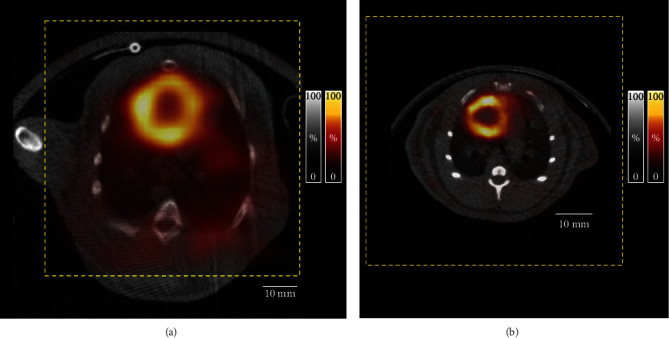
Relation between the field of view (FOV) and the body sizes of a rabbit and a rat. (a) Representative chest SPECT images with [^99m^Tc]Tc-MIBI (hot metal color) of a rabbit and (b) a rat using a GP-MSA collimator fused with a corresponding CT image (black and white color). The field of view in the single-bed position for SPECT imaging (yellow dot box) covers the heart and lung regions of the rabbit and rat.

*In vivo* SPECT images of the rabbit and rat are shown in Figures 4 and 5. The images of the whole lungs and heart of the rabbit were obtained without bed motion. Dynamic myocardial perfusion SPECT images of the investigated healthy rabbit were successfully generated (Figure 4(a)). The background activity gradually decreased, and cardiac uptake was clearly seen 3-5 min after injection. Time activity curves show the dynamic changes of cardiac and blood pool activity (Figure 4(b)). Imaging results were confirmed by SPECT of the second rabbit. Static SPECT images could be accurately fused with CT images (Figure 4(c)). The size of the GP-MSA collimator along with its substantially wide cFOV can easily cover the heart and lung regions of a rabbit (Figure 5(a)) and rat (Figure 5(b)).

## 4. Discussion

It is known that SPECT image quality is strongly influenced by collimator design. In this study, one of the first commercially available medium-sized animal GP-MSA collimators for high-resolution animal SPECT imaging systems was investigated using phantom and *in vivo* dynamic imaging in rabbits. The GP-MSA collimator achieved a high sensitivity of 790 cps/MBq, which is comparable with previously reported values for general-purpose mouse type (GP-M) collimators (847 cps/MBq) [19]. The spatial resolution was 2.5 mm, thereby indicating that the spatial resolution of collimators roughly corresponded to each aperture diameter [18, 19, 21, 32]. We included relatively low concentrations of 0.1 MBq/mL in our phantom experiments, because the amount of tracer that can be administered as well as the scan duration is limited in a busy *in vivo* animal experiment set-up with a high throughput.

In the CNR analysis, CNR values of 1.3 were achieved at rod sizes of 2.5 mm and larger. Rapid decrease was confirmed for rod sizes of 2.2 mm and smaller for all activity concentrations. This result was corresponding to the visual analysis of spatial resolution. Considering its performance, it seems to be impossible to obtain a spatial resolution of 2 mm and less using this collimator. The feature of this collimator is to achieve a wide cFOV instead of a high spatial resolution. Given that the weight of a rabbit is more than 30 times heavier than that of a mouse, and that each organ is more than three times as big, this collimator should be able to address the larger body size and habitus of rabbits.

Uniformity was 39.0%, slightly worse than the 29.1% of the GP-M collimator for the same scanner [19]. Direct comparison of those two collimators is not appropriate because the voxel size during reconstruction and FWHM in Gaussian postfiltering were different for each collimator. Furthermore, when using the GP-MSA collimator, a single bed position scan was used because the cFOV of the collimator is wide enough to cover the whole phantom, while multiple bed positions are needed for other collimators such as GP-M. To be noted, using GP-MSA, we found that the horizontal line profile appeared slightly more irregular when compared to the vertical one. This might be a characteristic artifact of scanners with two detectors and needs to be confirmed by direct comparison of two- and three-detector SPECT systems.

The advantage of the GP-MSA collimator is exclusively the wide cFOV. As shown in Figure 5(a), the size of its cFOV is 65 mm and thus sufficiently wide to scan the rabbits' thoraces. Generally, the disadvantage of pinhole collimators is a narrow cFOV. For example, the short-axis size of a rabbit heart is approximately 20 mm. The size of the cFOV of the collimator for rats is 28 mm [18], thereby insufficient to visualize any uptake of the rabbit's heart without bed motion. When qualitative evaluation is conducted in SPECT images, ratios of the target to the normal organs such as the blood pool and muscles are often used. The GP-MSA collimator allows to visualize almost the whole transaxial area of the rabbit and to analyze the target uptake as compared with radiotracer accumulation in other organs. The fact that we were able to perform dynamic imaging of rabbit thoracic organs in this study is an advancement for future SPECT imaging of rabbits. Future studies investigating performance of U-SPECT5-E/CT may also repeat the herein presented experiments using lower amounts of activities, as less injected MBq may also impact scanner accuracy. However, the required large amount of activity to achieve sufficient diagnostic accuracy may be also limitation for other studies, e.g., tumor-targeting radiopharmaceuticals that are highly susceptible to the current receptor expression.

## 5. Conclusions

We evaluated the performance of GP-MSA collimator on the U-SPECT5-E/CT for medium-sized animals such as rabbits. In this particular setting, the wide cFOV of the collimator was suitable for imaging medium-sized animals with SPECT in various purposes including dynamic *in vivo* assessments.

## Data Availability

All data is available on request to the corresponding author.

## Supplementary Materials

Supplementary Materials Supplementary Figure 1: design of a Derenzo type phantom for animal scanners (10111-21-2-008). Photographs of the phantom are displayed in the upper row, while lower images show the transaxial and coronal images of X-ray computed tomography. The phantom contains an acryl column whose height and width are 16 mm and 36 mm, respectively. The column has 6 different groups of rods whose diameters range from 1.80 to 3.10 mm. The distance between each lot is equal to the diameter of the respective rod in that section.
