# Supplementary material for: Performance Evaluation of a Preclinical SPECT Scanner with a Collimator Designed for Medium-Sized Animals
Source: Mol Imaging. 2022 Jul 16;2022:9810097. doi: 10.1155/2022/9810097 (PMC9328189; doi:10.1155/2022/9810097)
Supplement: Supplementary Materials — Supplementary Figure 1: design of a Derenzo type phantom for animal scanners (10111-21-2-008). Photographs of the phantom are displayed in the upper row, while lower images show the transaxial and coronal images of X-ray computed tomography. The phantom contains an acryl column whose height and width are 16 mm and 36 mm, respectively. The column has 6 different groups of rods whose diameters range from 1.80 to 3.10 mm. The distance between each lot is equal to the diameter of the respective rod in that section. [file 9810097.f1.docx]

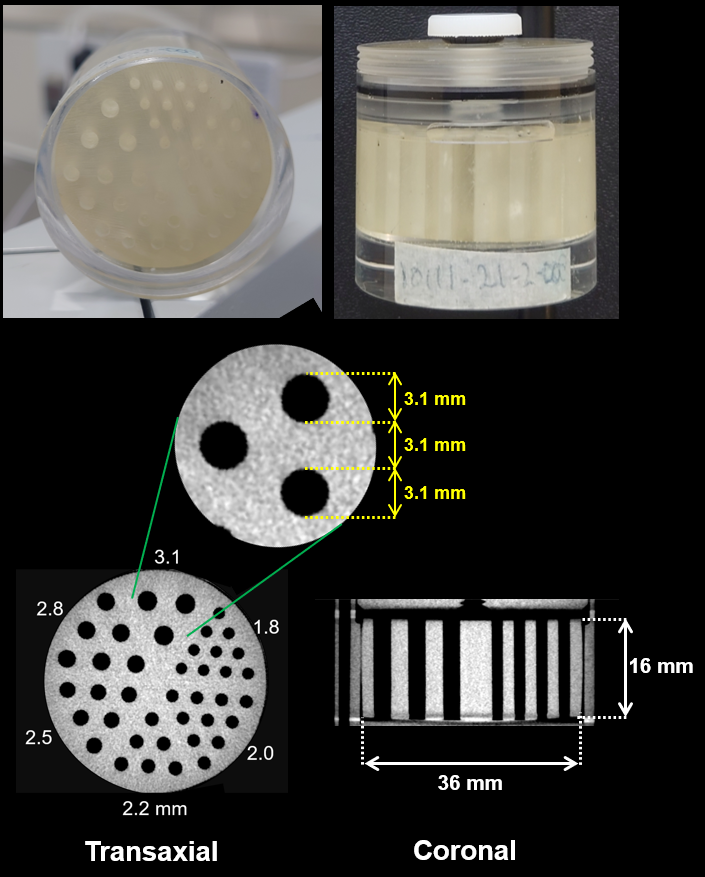


**Supplementary Figure 1**. Design of a Derenzo type phantom for animal scanners (10111-21-2-008). Photographs of the phantom are displayed in the upper row, while lower images show the transaxial and coronal images of X-ray computed tomography. The phantom contains an acryl column whose height and width are 16 mm and 36mm, respectively. The column has 6 different groups of rods whose diameters ranges from 1.80 to 3.10 mm. The distance between each lot is equal to the diameter of the respective rod in that section.
